# Synthesis and Characterization of High Entropy Alloy 23Fe-21Cr-18Ni-20Ti-18Mn for Electrochemical Sensor Applications

**DOI:** 10.3390/ma15217591

**Published:** 2022-10-28

**Authors:** Shashanka Rajendrachari, Vinayak Adimule, Mahir Gulen, Farshid Khosravi, Kiran Kenchappa Somashekharappa

**Affiliations:** 1 Department of Metallurgical and Materials Engineering, Bartin University, 74100 Bartin, Turkey; 2 Department of Chemistry, Angadi Institute of Technology and Management (AITM), Belagavi 590009, India; 3 Mechanical Engineering Department, Faculty of Engineering, Bartin University, 74100 Bartin, Turkey; 4 Department of Chemistry, Govt. First Grade College, Shivamogga 577201, India

**Keywords:** microstructure, ball milling, high entropy alloys, ascorbic acid, electrochemical sensors, anodic peak current

## Abstract

High entropy alloys (HEA) are one of the modern-era alloys accelerating with greater velocity because of their excellent properties and different applications. In the present paper, we have successfully fabricated HEA (23Fe-21Cr-18Ni-20Ti-18Mn) powders by ball milling the elemental Fe, Cr, Ni, Ti, and Mn powders for 15 h. The advancement of the milling process and phase transformation of HEAs were studied by using X-ray diffraction (XRD) and scanning electron microscope (SEM). The crystallite size and the lattice strain of the HEA were calculated by using the Williamson-Hall (W-H) equation and the values were found to be 7 nm and 0.0176%, respectively. Similarly, the true lattice parameter was calculated using the Nelson–Riley (N-R) extrapolation method, and the value was found to be 3.544 Å. We have successfully investigated the electrochemical response of 15 h ball milled 23Fe-21Cr-18Ni-20Ti-18Mn HEA powders to determine the ascorbic acid (AA) using cyclic voltammetry. We have modified the carbon paste electrode with ball milled HEA of concentrations 0, 2, 4, 6, 8, and 10 mg, and among them, 8 mg HEA modified carbon paste electrode (HEA-MCPE) depicted the highest current sensitivity. We reported the effect of modifier concentration, analyte concentration, scan rate, and pH on the oxidation peak of AA. The electrochemical active surface area of carbon paste and MCPE was calculated using the Nernst equation and the values were found to be 0.0014 cm^2^ and 0.0027 cm^2^, respectively. The fabricated HEA-MCPE showed excellent current sensitivity, stability, anti-fouling, and selectivity.

## 1. Introduction

**Figure 1:**
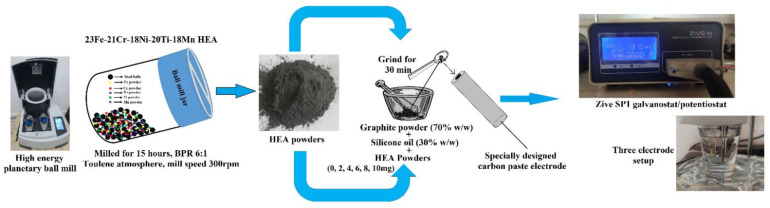
Graphical representation of the experiment.

People have been using metals and alloys for many centuries, starting from the civilization period. However, the world has witnessed an extraordinary improvement in alloys over the past few decades. Among them, refining the microstructure by changing the elemental composition, and improving and developing the new advanced ways of fabrication methods, have contributed tremendously to modifying the properties of alloys. The world has evolved from using bi and tri-metallic to multi-metallic alloys, which exhibit better properties than bi and tri-metallic alloys. These multi-metallic alloys consist of complex composition and show maximum mixing entropy than the mixing of entropy of bi, tri-metallic, or pure metals [1]. This high mixing entropy of HEA increases the enthalpy and allows for adding more alloying metals. Therefore, formulating any alloy with a specific and wide range of properties mainly depends upon the type of the manufacturing process, the type, and the number of alloying elements used. As the number of alloying elements increases, it will be a little difficult to understand their phase diagrams, but the parental alloy will exhibit excellent, unique properties due to the multi-phase structures. One such multi-phase alloy are HEAs, formed by mixing almost equal proportions of five or more metals together to change their properties significantly [2]. HEAs are alternatives to the traditional titanium, aluminum, copper, steel-based alloys. The HEAs are very popular due to their maximum toughness, high thermal and electrical conductivity, excellent creep, corrosion, and wear resistance properties. These properties of HEA can be further revised significantly by refining their microstructures and this can be easily achieved by planetary ball milling. The main advantage of using the planetary ball milling method is its ability to produce nano-structured alloys through solid solution formation with controlled properties [3,4,5]. It is possible to prepare any combination of alloys using ball milling methods, but quite difficult with other conventional metallurgical operations due to the differences in their physical properties. It is a type of powder metallurgy method used for the mass production of smaller objects with the complex shape at low cost. Figure 1 depicts the graphical representation of the experiment.

A German metallurgist, Franz Karl Archard, prepared HEA for the first time in the late 18th century by using an equal mass of five to seven elements; however, the results were not satisfactory [6]. Therefore, many researchers were not interested in preparing HEAs due to the failure of the HEA concept during that time. However, modern-day researchers have understood the importance of HEAs and are preparing a different variety of HEAs by changing the elements, composition, and fabricating methods. For the first time, Varalakshmi et al. (2008) [7] used the ball milling method to prepare AlCrCuFeTiZn HEAs, and they have been popularized the ball milling method of preparing HEAs ever since then. Table 1 depicts the various researchers who prepared different HEAs by the ball milling method.

In the present article, we have prepared 23Fe-21Cr-18Ni-20Ti-18Mn HEA using a planetary ball mill for 15 h and studied their morphology. The 15 h milled HEA powder was further used as a modifier in the bare carbon paste electrode (BCPE) and we studied their electrical response during the detection of AA.

AA is popularly named as vitamin C; a natural antioxidant, and used to improve our immunity, preventing cancer, etc. [18]. The deficiency of vitamin C can affect our immunity and cause scurvy, gingival bleeding, etc. Similarly, more consumption of AA can cause urinary stones, stomach convulsions, and diarrhea [19]. Therefore, vitamin C levels must be maintained properly or the periodic determination of AA must be performed. In this article, we have fabricated HEA-MCPE to determine AA using cyclic voltammetry (CV). This method is easy, accurate, and cost-effective, compared to other methods. For more details about cyclic voltammetry, it is recommended to refer to the author’s previous publications [20,21,22,23]. Many researchers have published research articles on the electrochemical determination of AA by the cyclic voltammetry method, using various modifiers such as nanomaterials, surfactants, polymers, and carbon nanotubes, etc. [24,25,26,27]. However, very few articles reported the use of HEAs, such as 23Fe-21Cr-18Ni-20Ti-18Mn, as a modifier in the BCPE to determine AA and related bio-molecules as tabulated in Table 2. The present fabricated HE-MCPE exhibited excellent current sensitivity, selectivity, and stability in determining AA in low concentrations.

## 2. Materials and Methods

### 2.1. Fabrication of 23Fe-21Cr-18Ni-20Ti-18Mn HEA

The elemental powder mixture of Fe (99.6% pure), Cr (99.8% pure), Ti (99.6% pure), Ni (99.5% pure), and Mn (99.2% pure) was used as precursors, and the composition of 23Fe-21Cr-18Ni-20Ti-18Mn HEA powder was prepared by using Retsch Planetary Ball Mill PM 100 purchased from Retsch GmbH, Haan, Germany. The milling was performed for 15 h with a mill speed of 300 rpm under a toluene atmosphere to prevent oxidation. The BPR was maintained at 6:1, and the chrome steel milling jar of volume 500 mL and the 10 mm diameter chrome steel balls are used as a milling media. Before starting the milling, it was important to make sure all the balls are completely immersed in the toluene. At regular intervals of time (0, 2, 5, 10, and 15 h of ball milling), a small amount of the HEA powder was taken out for further characterization by XRD and SEM. The phase analysis was performed using RIGAKU smart lab TM X-Ray diffractometer purchased from Rigaku Europe SE, Neu-Isenburg, Germany. The SEM (TESCAN MAIA3 XMU) purchased from TESCAN ORSAY HOLDING, Brno-Kohoutovice, Czech Republic was used to study the morphology of the HEA powders and EDS attached to SEM was used for elemental analysis of the ball milled HEA powders.

### 2.2. Fabrication of the Carbon Paste Electrodes

The Zive SP1 galvanostat/potentiostat electrochemical system was used to study the electrochemical sensor applications of ball milled 23Fe-21Cr-18Ni-20Ti-18Mn HEA. We have used a working electrode (carbon paste electrode of 3 mm diameter), a reference electrode (Ag/AgCl saturated KCl), and a counter electrode (platinum wire). The fabrication of homogeneous BCPE is standard and reported by the authors in their previous publications [18,20,22]. The fabricated paste was then closely inserted into a 3 mm cavity of a polymer tube attached to a copper wire for the sake of electrical contact (acts as a working electrode). On the other hand, the different concentration of the ball milled HEA modifier (2, 4, 6, 8, and 10 mg) was hand mixed separately with graphite powder and silicon oil at a ratio of 70:30 (*w*/*w*) in an agate mortar for 30 min.

## 3. Results

### 3.1. X-ray Diffraction (XRD)

**Figure 2:**
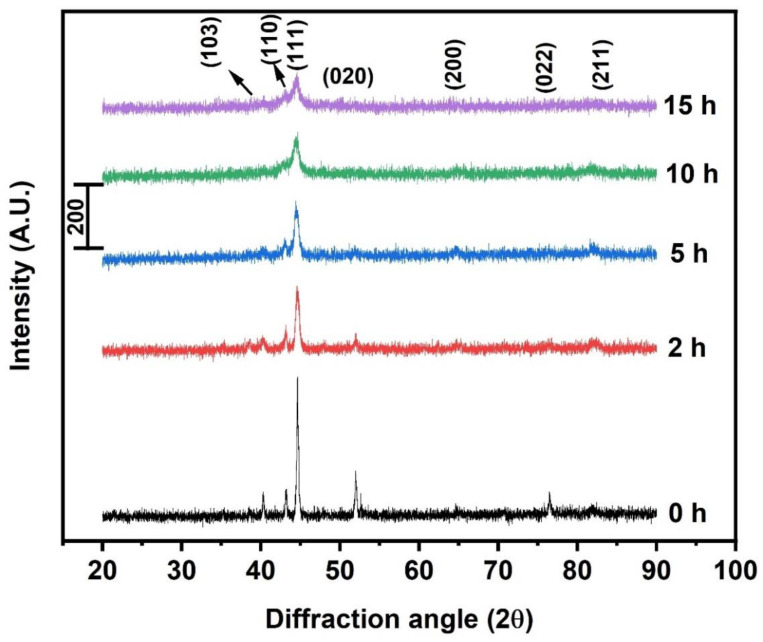
XRD spectra of 23Fe-21Cr-18Ni-20Ti-18Mn HEA powders milled at different time intervals.

Figure 2 depicts the XRD spectra of the elemental composition of 23Fe-21Cr-18Ni-20Ti-18Mn HEA powders milled at 0, 2, 5, 10, and 15 h. At 0 h (before milling), we can see the sharp and narrow crystalline diffraction peaks of the elemental composition of HEA corresponding to the pure elements. As milling starts, peak intensity starts reducing and peak broadening will take place. The diffraction peaks corresponded to Ni-Ti (110) and (210) at an angle of 43.80° and 70.98° and start disappearing with the milling time, before completely disappearing at 15 h of milling. The disappearance of these peaks is due to the solid solution formation as a result of which the Ni, and Ti go into interstitial sites of Fe. As milling continues, most of the elements dissolve into Fe lattice, and aid solid solution formation at 10 h of milling. Even though solid solution formation was reached after 10 h of ball milling, we continued the milling to homogenize the solid solution of refined crystallites. Similarly, the Mn-Ti (103) peak at a diffraction angle of 40.38° undergoes broadening and reduces its intensity. The formation of the solid solution phase may mainly depend upon the high entropy and a strong bonding among the constituent elements, their differences in atomic sizes, etc.

We can observe the slight shifting of diffraction angle of (111) towards lower values due to the residual stress accumulated because of the high impact generated during ball milling. Sometimes, the reduced crystallite size or change in the lattice parameter also results in shifting of diffraction peaks [34]. The peak broadening and the gradual decrease in the intensity of the fabricated HEA powders with respect to milling time are due to the increased lattice strain and reduced crystallite size [35,36,37]. As a result of this, amorphization and structural defects occur and thus volume fraction of grain boundaries increases with reduced crystallite size with the milling. That phenomenon increases the defect storage sites, reduces the path of diffusion, and reaches a meta-stable non-equilibrium state. Therefore, the fabricated HEA powders exhibit refined crystallite size, increased dislocations, and strain.

#### 3.1.1. Crystallite Size and Lattice Strain Calculation by W–H Method

As we discussed earlier, the diffraction peak broadening is due to several factors, such as refined crystallite size, instrumental errors, and increased lattice strain. Generally, the lattice strain and the crystallite size vary significantly with the milling time [34]. Therefore, we determined them for the 23Fe-21Cr-18Ni-20Ti-18Mn HEA at different milling times of 0, 2, 5, 10, and 15 h, using the W-H equation. This method involves de-convoluting the crystallite size and lattice strain by measuring the peak width and thus minimizing the peak broadening errors. The authors have explained the W-H method in their previous publication [3,4] and the equation is as follow:

(1) $$\beta \;cos\theta =\frac{0.94\lambda }{D}+4\eta sin\theta$$

where *β* width half maxima (FWHM), *D* crystallite size and *η* lattice strain.

**Figure 3:**
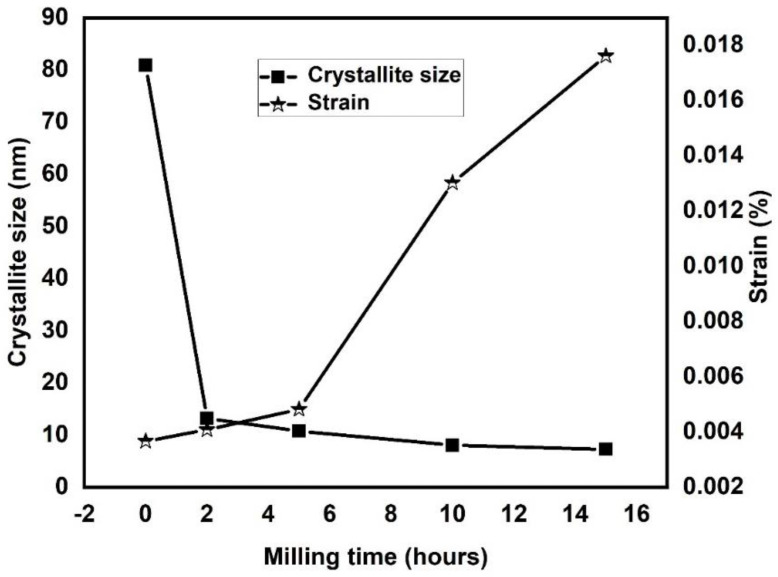
Graphical representation of crystallite size and lattice strain of HEA at different milling time.

The crystallite size and the lattice strain of 23Fe-21Cr-18Ni-20Ti-18Mn HEA powders milled at different milling intervals are shown in Figure 3. It is noticed that, as the milling time of the HEA powders increases, the crystallite size decreases, and the lattice strain increases. After 5 h of milling, the crystallite size of fabricated HEA does not reduce much and reaches saturation. Milling after 5, 10, and 15 h does not affect the crystallite size significantly, but the lattice strain kept increasing, as shown in the graph, due to the continuous contact of powder, balls, and jar surface. Because of the strong interaction of the ball, powder, and the jar, the crystallite size of 23Fe-21Cr-18Ni-20Ti-18Mn HEA powders reduces from 81 nm to 7 nm after a milling time from 0 to 15 h. Similarly, lattice strain increases from 0.003% to 0.017% from 0 to 15 h milling time, respectively.

#### 3.1.2. Lattice Parameter Calculation by N–R Extrapolation Method

The peak broadening in XRD may affect the actual lattice parameter and, therefore, the N–R extrapolation method was used to calculate the true lattice parameter of ball-milled HEA powders by reducing the instrumental errors. In this method, the N–R functions were calculated for three strong diffraction peaks below the equation [34].

(2) $$\frac{cos^{2}\theta }{sin\theta }+\frac{cos^{2}\theta }{\theta }$$

The true lattice parameter of 23Fe-21Cr-18Ni-20Ti-18Mn HEA powders was calculated by considering the diffraction peaks corresponding to (111), (020), and (022) planes, respectively, for 0, 2, 5, 10, and 15 h milled samples. On the other hand, we calculated the lattice parameter of the same diffraction peaks at the same milling time using the equation as follows:

(3) $$a=d\sqrt{h^{2}+k^{2}+l^{2}}$$

**Figure 4:**
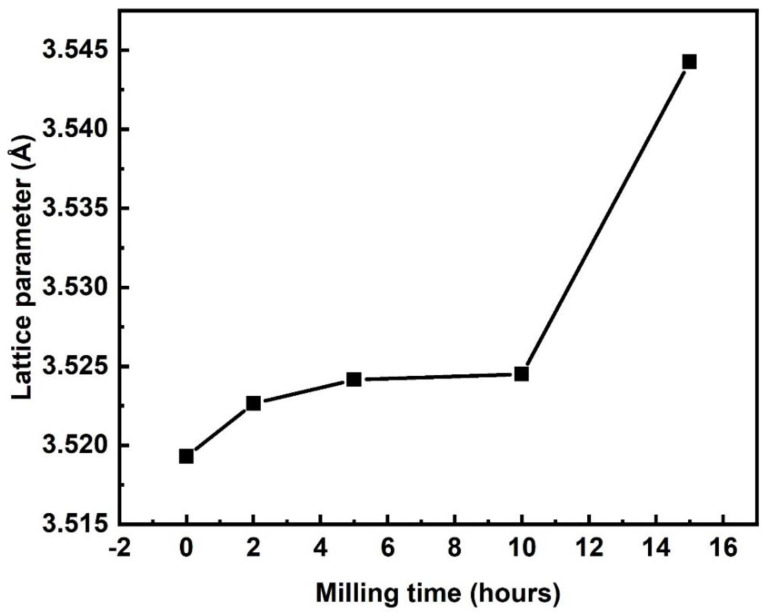
Variation of the true lattice parameter of 23Fe-21Cr-18Ni-20Ti-18Mn HEA powders with respect to milling time.

Considering Equation (2) as the *x*-axis and Equation (3) as the *y*-axis, their respective values were fitted linearly in a straight line and extrapolated to the *y*-axis. The point of intersection on the *y*-axis gives the actual lattice parameter value with filtered instrumental errors. Figure 4 depicts the graph of the true lattice parameter of 23Fe-21Cr-18Ni-20Ti-18Mn HEA powders derived from the N–R method. It was noticed that the lattice parameter increases from 3.519 to 3.544Å with a milling time from 0 to 15 h, respectively. The true lattice parameter of fabricated 23Fe-21Cr-18Ni-20Ti-18Mn HEA powders after 15 h of ball milling is 3.544Å. The increased lattice spacing with increased milling time is due to the huge amount of defects formed during milling, and also the diffusion of Mn, Ni, Ti, and Cr elements into the lattice of Fe atoms.

### 3.2. Investigation of the Microstructure of 23Fe-21Cr-18Ni-20Ti-18Mn HEA Powders

**Figure 5:**
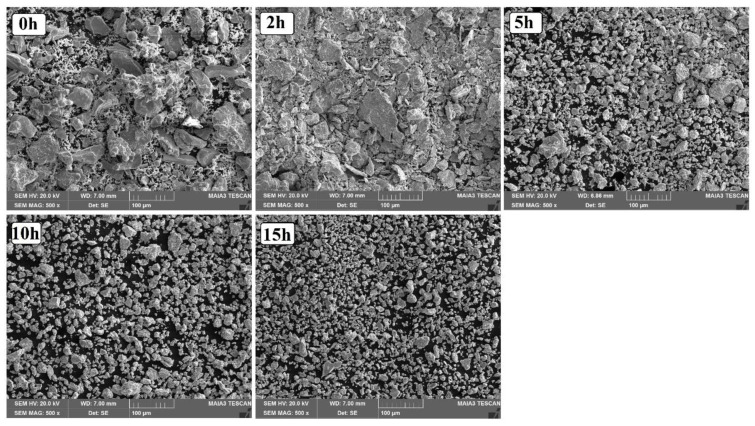
SEM of 0, 2, 5, 10, and 15 ball-milled 23Fe-21Cr-18Ni-20Ti-18Mn HEA powders.

Figure 5 represents the SEM microstructures of 23Fe-21Cr-18Ni-20Ti-18Mn HEA powders ball milled at 0, 2, 5, 10, and 15 h, respectively. It was observed that at 0 h the elemental powders of HEA are huge and exhibit random shapes. As milling starts, the elemental powders begin to collide by jar-balls-powders and undergo flattening due to the ductile nature of Fe. At 2 h of milling, a few flattened surfaces combine together and grow in size, as shown in the figure. Further milling decreases the flat surface spacing and the brittle elements Cr, Ni, Ti, and Mn get uniformly dispersed on the flat surface. However, after 5 h, the particles will undergo plastic deformation or work hardening and all the elements go into the solid solution of Fe atoms. The same mechanism will continue untill 10 h of milling, and all the elements are distributed uniformly and form a homogenized alloy. Even though a solid solution formed at 10 h of milling, we still continued the milling untill 15 h to refine the particle size. Refining the particle size enhances the surface area and surface energy, which is very much essential to study their electrochemical applications [20,32,33]. It has been found that 15 h of milling resulted in partial spherical particles with a reduced particle size, compared to 10 h of milling. The average particle size of 15 h ball milled 23Fe-21Cr-18Ni-20Ti-18Mn HEA powders was found to be 5–7 μm.

### 3.3. Energy Dispersive Spectroscopy (EDS) and Elemental Mapping

**Figure 6:**
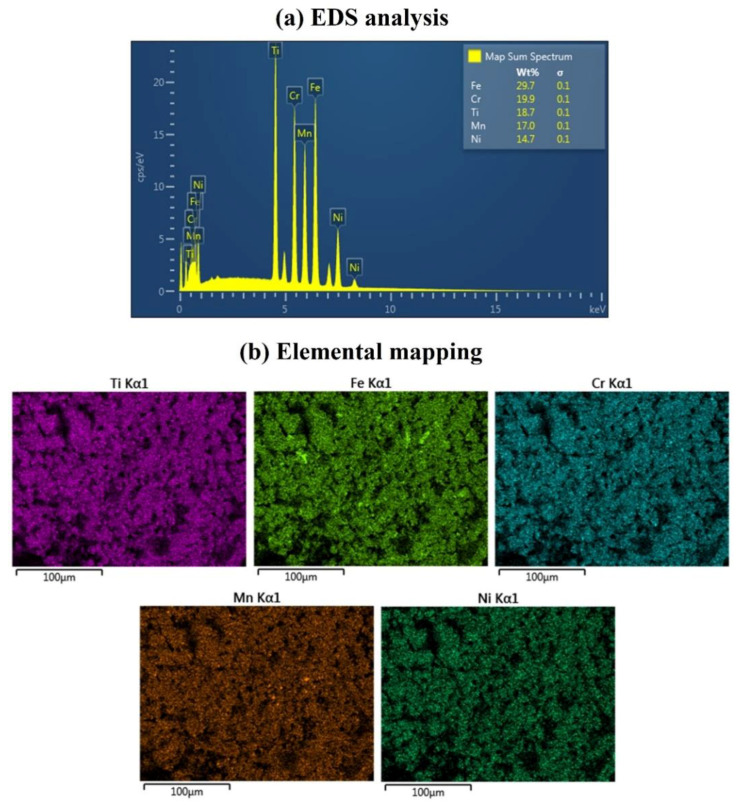
Elemental analysis and their distribution in 15 h ball milled HEA powders.

Semi-quantitative technique elemental studies of fabricated 23Fe-21Cr-18Ni-20Ti-18Mn HEA were performed using EDS attached to SEM. Figure 6a shows the EDS spectrum of 15 h milled HEA powders. The EDS analysis confirms the presence of all the parental elements used to prepare HEA powders; there is no impurity and oxidation found in the alloys. The prepared HEA is highly pure and shows a slightly increased amount of Fe (Wt.%) compared to the parental composition. This is due to the diffusion of Ti, Ni, Cr, and Mn into the lattice of Fe. The EDS analysis confirms that the chemical composition of 15 h milled HEA powders is closely related to the elemental composition of the HEA powders before milling. The inset of EDS (Figure 6a) has the chemical composition of the 15 h ball milled HEA powders. We also studied the elemental distribution in the alloy using elemental mapping software during EDS analysis. Figure 6b depicts the solid solution distribution of Fe, Cr, Ni, Ti, and Mn after 15 h of ball milling. The fabricated 23Fe-21Cr-18Ni-20Ti-18Mn HEA exhibits the uniform distribution of the elements with no impurity.

### 3.4. Electrochemical Detection of AA Using HEA-MCPE

#### 3.4.1. Optimizing the Concentration of the Modifier (HEA) to Detect AA

To achieve maximum current sensitivity, one must take care of many parameters; one among them is the concentration of the modifier in the carbon paste. The fabricated modifier exhibits a larger surface area due to the extreme work hardening and refining during ball milling. Most of the transition elements show excellent current sensitivity and it is reported by many authors [32,33]. Our HEA has a total of five transition elements, which are responsible for the excellent sensitivity. Very few papers reported the use of alloys as electrochemical sensors [20,32,33]. We are the first to report the electrocatalytic applications of 21Cr-18Ni-20Ti-18Mn HEA-MCPE to determine AA. In the present paper, we have used 23Fe-21Cr-18Ni-20Ti-18Mn HEA-MCPE to determine AA. We have prepared the different concentrated MCPEs (2, 4, 6, 8, 10 mg) and recorded their anodic peak currents against AA, and compared their results with the bare carbon paste electrode (BCPE), in other words, 0 mg MCPE.

**Figure 7:**
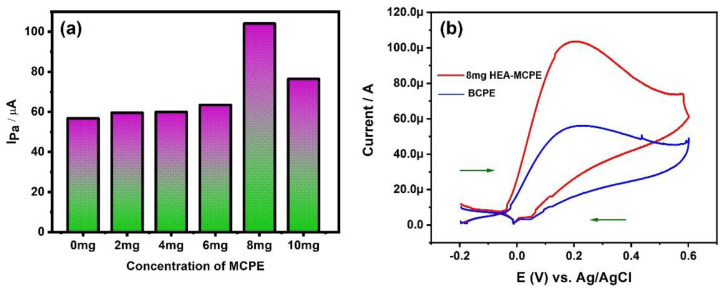
(**a**) Graphical representation of anodic peak current of HEA-MCPE at different concentrations (0, 2, 4, 6, 8, and 10 mg). (**b**) Cyclic voltammogram (CV) of 2 mM AA at BCPE and 8 mg HEA-MCPE.

Figure 7a is the graphical representation of different concentrations of the ball milled HEA modifier with Ipa. The graph shows the increased Ipa up to 8 mg HEA modifier and decreases after that. The maximum peak current of 104.07 µA was obtained for the concentration of 8 mg modifier. The increased current sensitivity for 8 mg HEA-MCPE is due to the increased number of oxidation sites and the electrode area [38]. Therefore, we have used 8 mg HEA-MCPE for studying the electrochemical properties further. The ball milled HEA modifier not only increases the Ipa but also reduces the overpotential. Figure 7b depicts the CV curve of 2 mM AA at BCPE and 8 mg HEA-MCPE. It is observed from the voltammogram that the anodic peak current of BCPE is lower than HEA-MCPE, and the values were found to be 56.7 µA and 104.07 µA, respectively. We also calculated the active surface area of HEA-MCPE using the equation below [39]:

i_p_ = 2.69 × 10^5^ n^3/2^AD^1/2^Cν^1/2^ (4)

The details of the calculation of the electrode surface area and the terminology of the equation were well discussed by the authors in their previous paper [39]. The calculated active surface area for the electron transfer process of AA for BCPE and HEA-MCPE are 0.0014 cm^2^ and 0.0027 cm^2^, respectively.

#### 3.4.2. Effect of Scan Rate

**Figure 8:**
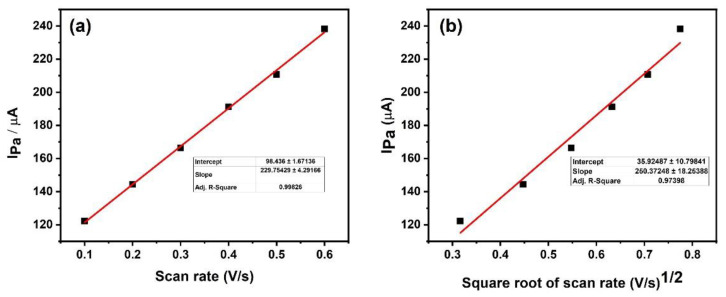
(**a**) The plot of Ipa vs. scan rate, and (**b**) Ipa vs. square root of scan rate.

We have studied the effect of scan rate on the intensity of the anodic peak current to understand whether the electrochemical reaction is diffusion or adsorption controlled. We have studied the anodic peak current of AA by varying the scan rate from 100 to 600 mV/s, in a phosphate buffer solution (PBS) of pH 7.2. Figure 8a depicts the plot of Ipa vs. scan rate and Figure 8b depicts the Ipa vs. square root of scan rate. From the graph, it is clear that an increase in the scan rate from 100 to 600 mV increases the Ipa linearly due to the direct electron transfer between AA and the HEA-MCPE surface. The correlation coefficient of Ipa versus the scan rate and Ipa versus square root of scan rate was determined to be 0.9982 and 0.9739, respectively. The value of Ipa vs scan rate is almost equal to 1. Therefore, the mass transfer process is diffusion-controlled.

#### 3.4.3. Effect of pH

**Figure 9:**
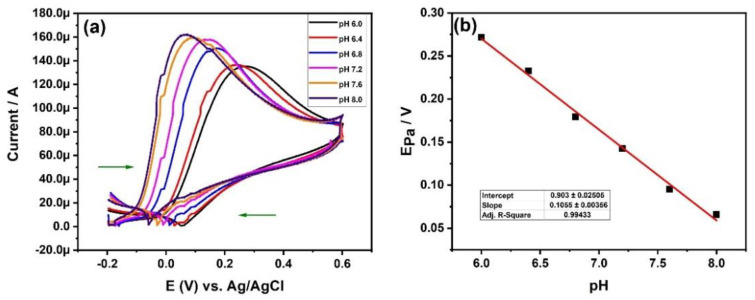
(**a**) Cyclic voltammogram of HEA-MCPE in 2 mM AA solution at different pH with a scan rate of 100 mVs^−1^. (**b**) Graph of pH vs. Epa at 2 mM AA.

The selection of pH plays an important role in deciding the current sensitivity of the particular electrode. Therefore, the optimization of pH must be performed to determine the optimum pH where the current sensitivity is maximum, and also to gain a better understanding of the transfer of the number of electrons and protons [40]. Figure 9a represents the cyclic voltammogram of HEA-MCPE in 2 mM AA solution at different pH (6, 6.4, 6.8, 7.2, 7.6, and 8.0) with a scan rate of 100 mVs^−1^. A cyclic voltammogram confirms that the anodic peak current increased with an increase in pH; whereas the oxidation peaks potential shift towards the lower values with an increase in pH, as shown. The cyclic voltammogram at pH 7.6, and 8.0 shifted to very low electrode potential, therefore optimum pH for the AA determination using HEA-MCPE is 7.2. We performed all the cyclic voltammetric investigations at pH 7.2. Figure 9b depicts the plot of pH vs Epa oxidation peak potential at 2 mM AA. From the plot, it is confirmed that the Epa decreases linearly from pH 6.0 to 8.0 with a correlation coefficient of 0.9943.

#### 3.4.4. Effect of Variation in DA Concentration

**Figure 10:**
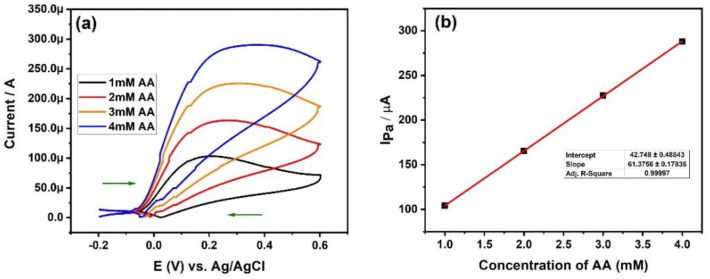
(**a**) CV curve of AA at different concentrations. (**b**) Plot of Ipa vs. the concentration of AA.

The concentration of the analytes plays an important role in the current sensitivity of the electrode. Generally, as the concentration of the analytes increases, the current response also increases linearly [41,42,43]. This is due to the availability of more numbers of analyte molecules [44]. We have investigated the effect of AA concentration using fabricated HEA-MCPE at a scan rate of 100 mVs^-1^. We collected the cyclic voltammogram at 1, 2, 3, and 4 mM of AA concentration using HEA-MCPE, as shown in Figure 10a. From the figure, it is confirmed that the increase in the concentration of AA has increased the anodic peak current linearly, as shown. Figure 10b depicts the plot of Ipa against the concentration of AA. The linear increase of Ipa is due to the successive increase in the number of AA molecules at the interface of fabricated HEA-MCPE. The correlation coefficient was calculated to be 0.9999. Refer Table 2 for the comparison of our results with the different types of alloy modified electrodes, as reported by various researchers.

## 4. Conclusions

We have successfully prepared the 23Fe-21Cr-18Ni-20Ti-18Mn HEA using a planetary ball mill for 15 h. We studied the phases, crystallite size, lattice strain, and lattice parameters using XRD spectra. The crystallite size decreases, but the lattice parameter and lattice strain increase, with a milling time from 0 to 15 h. The particle morphology and the microstructural evolution were successfully discussed using SEM. The qualitative, quantitative analysis was performed using EDS, whereas, the elemental distribution in the solid solution HEA was studied by elemental mapping. The fabricated HEA powders were refined using ball milling and further used as a modifier in the carbon paste electrode to determine the AA at a lower concentration. The fabricated HEA-MCPE showed a strong electrocatalytic activity towards the oxidation of AA. The 8 mg concentrated HEA-MCPE has shown maximum current response compared to 2, 4, 6, and 10 mg HEA. We further studied the effect of scan rate, pH, and the concentration variation of the analyte (AA) using 8 mg HEA-MCPE. We also calculated the electrode surface area for both BCPE and MCPE, where the MCPE surface area is almost double the surface area of BCPE. The fabricated HEA-MCPE has shown excellent sensitivity and selectivity in determining AA. The present electrode can be a potential sensor in the medical field for the diagnosis of AA deficiency diseases. The electrode reactions during the electrochemical oxidation of AA are diffusion controlled.

## Figures and Tables

**Table 1:** Depicts the various researchers who prepared different HEAs for the ball milling method.

Composition of HEA Used	Type of Ball Mill Used	Milling Parameters	Milling Result	References
AlCoCrFeNi	Planetary ball mill	Mill speed of 600 rpm for 60 h, ball-to-powder ratio (BPR) 10:1 under Argon atmosphere	Irregular shape with fractured surfaces, the average particle size of 10 µm	[8]
CoCrFeNiMo_0.85_	Pulverisette 6 Classic Line planetary ball mill	Milled for 30 h, N-Heptane process controlling agent (PCA), 350 rpm mill speed, BPR 10:1 under argon atmosphere	Irregular shape with fractured surfaces, the average particle size of 15 µm, no impurity	[9]
NbMoTaWVTi	High-energy planetary ball mill	Milled for 40 h, BPR 13:1 under Argon atmosphere	The average particle size is less than 10 µm, irregular shape of powders	[10]
CoCrFeNi	Pulverisette 6 ball mill	300 rpm mill speed, 50 h mill time, argon atmosphere, 10:1 BPR	Impurity (carbon and oxygen content) increased with the milling time	[11]
CoCrNiCuZn	Planetary ball mill	Milled at 300 rpm for 60 h under argon atmosphere, BPR 20:1, N-heptane as PCA	Crystallite size of 13 nm and lattice strain of 0.70%, irregular elliptical shape with a particle size of 3 µm	[12]
CuNiCoZnAlTi	Fritsch Pulverisette P-5 planetary ball mill	BPR 10:1, 300 rpm mill speed, toluene as PCA, 20 h of milling	Crystallite size of 9 nm and the lattice strain of 1.47%, the entropy of fusion is 11.5 J/mol K)	[13]
CoCrFeNiMo0.14Nb0.14	Fritsch P5 high energy ball mill	BPR 10:1, toluene as PCA, 30 h of milling	Mo diffuses very slowly in the HEA due to its high melting point	[14]
Al0.3NbTa0.8Ti1.5V0.2Zr	Fritsch Pulverisette 6 planetary ball mill	50 h milling, 10:1 BPR, 250 rpm speed under argon atmosphere	BCC crystal structure, irregular but elongated, the average particle size of 15 to 20 µm	[15]
Al0.2Co1.5CrFeNi1.5Ti	Fritsch Pulverisette 6 planetary ball mill	BPR 10:1, mill speed 300 rpm, 5 h of milling under nitrogen atmosphere	Uniform, fine-grained Microstructure, FCC crystal structure	[16]
CoCrFeNiMnAl	Planetary ball mill	60 h milled at 250 rpm in an argon atmosphere, BPR 15:1, N-heptane as PCA	15 nm crystallite size and 0.69% lattice strain, partially spherical with less than 40 nm particle size	[17]
23Fe-21Cr-18Ni-20Ti-18Mn	Retsch Planetary Ball Mill PM 100	15 h milled at 300 rpm under toluene atmosphere, BPR 6:1	Crystallite size of 7 nm and lattice strain 0.017%, irregular shape	[This paper]

**Table 2:** Comparison of our results with the different types of alloy modified electrodes, as reported by various researchers.

Composition of Alloy	Equipment Used	Analyte Used	Important Findings	References
NiFeCrCoCu	Cyclic voltammetry	Urea	Composition of 70:30 of HEA and the graphene showed better current sensitivity of 37.4 µAmM^−1^cm^−2^ towards the oxidation of urea than 50:50 and 90:10 composition	[28]
NiCrCuCoFe	Cyclic voltammetry	Glucose	The 50:50, and 70:30 composition of HEA and graphites mixture depicted better current sensitivity towards the oxidation of Glucose	[29]
10Pt-10Ti-80Al	Cyclic voltammetry	Ascorbic acid	The alloy was successfully employed to determine the ascorbic acid and also simultaneous detection of dopamine, ascorbic acid and uric acid was reported	[30]
PdPt	Cyclic voltammetry	Ascorbic acid	The alloy showed excellent sensitivity of 467.9 mAmM^−1^cm^−2^ towards the electroxidation of AA	[31]
Fe-18Cr-13Ni	Cyclic voltammetry	Folic acid	Electrode reactions were adsorption controlled. Current sensitivity of 17.32 µA was recorded	[20]
Duplex stainless steel	Cyclic voltammetry	Ascorbic acid	The stainless steel showed good current sensitivity of 143.52 µA	[32]
Duplex stainless steel	Cyclic voltammetry	Uric acid	The stainless steel showed good current sensitivity of 19.36 µA	[32]
Duplex stainless steel	Cyclic voltammetry	Dopamine	The stainless steel showed good current sensitivity of 25.61 µA	[32]
Yttria dispersed Fe-18Cr-13Ni	Cyclic voltammetry	Ascorbic acid	The stainless steel showed good current sensitivity of 370 mV	[33]
Yttria dispersed Fe-18Cr-13Ni	Cyclic voltammetry	Uric acid	The stainless steel showed good current sensitivity of 31.01 µA	[33]
Yttria dispersed Fe-18Cr-13Ni	Cyclic voltammetry	Dopamine	The stainless steel showed good current sensitivity of 28.48 µA	[33]
23Fe-21Cr-18Ni-20Ti-18Mn	Cyclic voltammetry	Ascorbic acid	The current sensitivity of 104.07 µA was obtained for the electrochemical oxidation of AA. The electrochemical active surface area of MCPE was found to be 0.0027 cm^2^	[Present paper]

## References

[1] Rajendrachari S. (2022). An Overview of High-Entropy Alloys Prepared by Mechanical Alloying Followed by the Characterization of Their Microstructure and Various Properties. Alloys.

[2] Wu Y., Liaw P.K., Zhang Y. (2021). Preparation of Bulk TiZrNbMoV and NbTiAlTaV High-Entropy Alloys by Powder Sintering. Metals.

[3] Shashanka R., Chaira D. (2014). Phase transformation and microstructure study of nano-structured austenitic and ferritic stainless steel powders prepared by planetary milling. Powder Technol..

[4] Shashanka R., Chaira D. (2015). Development of nano-structured duplex and ferritic stainless steel by pulverisette planetary milling followed by pressureless sintering. Mater. Charact..

[5] Gupta S., Shashanka R., Chaira D. (2015). Synthesis of nano-structured duplex and ferritic stainless steel powders by planetary milling: An experimental and simulation study. IOP Conf. Ser. Mater. Sci. Eng..

[6] Tokarewicz M., Grądzka-Dahlke M. (2021). Review of Recent Research on AlCoCrFeNi High-Entropy Alloy. Metals.

[7] Varalakshmi S., Kamaraj M., Murty B.S. (2008). Synthesis and characterization of nanocrystalline AlFeTiCrZnCu high entropy solid solution by mechanical alloying. J. Alloys Compd..

[8] Arab A., Guo Y., Zhou Q., Chen P. (2019). Fabrication of Nanocrystalline AlCoCrFeNi High Entropy Alloy through Shock Consolidation and Mechanical Alloying. Entropy.

[9] Geambazu L.E., Cotruţ C.M., Miculescu F., Csaki I. (2021). Mechanically Alloyed CoCrFeNiMo0.85 High-Entropy Alloy for Corrosion Resistance Coatings. Materials.

[10] Long Y., Su K., Zhang J., Liang X., Peng H., Li X. (2018). Enhanced Strength of a Mechanical Alloyed NbMoTaWVTi Refractory High Entropy Alloy. Materials.

[11] Moravcik I., Kubicek A., Moravcikova-Gouvea L., Adam O., Kana V., Pouchly V., Zadera A., Dlouhy I. (2020). The Origins of High-Entropy Alloy Contamination Induced by Mechanical Alloying and Sintering. Metals.

[12] Sun Y., Ke B., Li Y., Yang K., Yang M., Ji W., Fu Z. (2019). Phases, Microstructures and Mechanical Properties of CoCrNiCuZn High-Entropy Alloy Prepared by Mechanical Alloying and Spark Plasma Sintering. Entropy.

[13] Varalakshmi S., Kamaraj M., Murty B.S. (2010). Processing and properties of nanocrystalline CuNiCoZnAlTi high entropy alloys by mechanical alloying. Mater. Sci. Eng. A.

[14] Praveen S., Murty B.S., Kottada R.S. (2014). Effect of Molybdenum and Niobium on the Phase Formation and Hardness of Nanocrystalline CoCrFeNi High Entropy Alloys. J. Nanosci. Nanotechnol..

[15] Moravcikova-Gouvea L., Moravcik I., Pouchly V., Kovacova Z., Kitzmantel M., Neubauer E., Dlouhy I. (2021). Tailoring a Refractory High Entropy Alloy by Powder Metallurgy Process Optimization. Materials.

[16] Moravcikova-Gouvea L., Moravcik I., Omasta M., Veselý J., Cizek J., Minárik P., Cupera J., Záděra A., Jan V., Dlouhy I. (2020). High-strength Al0.2Co1.5CrFeNi1.5Ti high-entropy alloy produced by powder metallurgy and casting: A comparison of microstructures, mechanical and tribological properties. Mat. Charact..

[17] Wang C., Ji W., Fu Z. (2014). Mechanical alloying and spark plasma sintering of CoCrFeNiMnAl high-entropy alloy. Adv. Powder Technol..

[18] Shashanka R., Jayaprakash G.K., Prakashaiah B.G., Kumar M., Swamy B.E.K. (2022). Electrocatalytic determination of ascorbic acid using a green synthesised magnetite nanoflake modified carbon paste electrode by cyclic voltammetric method. Mater. Res. Innov..

[19] Hu G., Guo Y., Xue Q., Shao S. (2010). A highly selective amperometric sensor for ascorbic acid based on mesopore-rich active carbon modifed pyrolytic graphite electrode. Electrochim. Acta.

[20] Shashanka R., Chaira D., Swamy B.E.K. (2015). Electrocatalytic Response of Duplex and Yittria Dispersed Duplex Stainless Steel Modified Carbon Paste Electrode in Detecting Folic Acid Using Cyclic Voltammetry. Int. J. Electrochem. Sci..

[21] Rajendrachari S., Swamy B.E.K., Reddy S., Chaira D. (2013). Synthesis of Silver Nanoparticles and their Applications. Anal. Bioanal. Electrochem..

[22] Shashanka R., Swamy B.E.K. (2020). Biosynthesis of silver nanoparticles using leaves of Acacia melanoxylon and its application as dopamine and hydrogen peroxide sensors. Phys. Chem. Res..

[23] Jayaprakash G.K., Swamy B.E.K., Rajendrachari S., Sharma S.C., Flores-Moreno R. (2021). Dual descriptor analysis of cetylpyridinium modified carbon paste electrodes for ascorbic acid sensing applications. J. Mol. Liq..

[24] Manjunatha J.G., Deraman M., Basri N.H., Mohd Nor N.S., Talib I.A., Ataollahi N. (2014). Sodium dodecyl sulfate modified carbon nanotubes paste electrode as a novel sensor for the simultaneous determination of dopamine, ascorbic acid, and uric acid. Comptes Rendus Chim..

[25] Manjunatha J.G., Deraman M. (2017). Graphene Paste Electrode Modified with Sodium Dodecyl Sulfate Surfactant for the Determination of Dopamine, Ascorbic Acid and Uric Acid. Anal. Bioanal. Electrochem..

[26] Manjunatha J.G., Deraman M., Basri N.H., Talib I.A. (2018). Fabrication of poly (Solid Red A) modified carbon nano tube paste electrode and its application for simultaneous determination of epinephrine, uric acid and ascorbic acid. Arab. J. Chem..

[27] Shankar S.S., Swamy B.E.K., Chandra U., Manjunatha J.G., Sherigara B.S. (2009). Simultaneous Determination of Dopamine, Uric Acid and Ascorbic Acid with CTAB Modified Carbon Paste Electrode. Int. J. Electrochem. Sci..

[28] Ashwini R., Punith Kumar M.K., Rekha M.Y., Santosh M.S., Srivastava C. (2022). Optimization of NiFeCrCoCu high entropy alloy nanoparticle-graphene (HEA-G) composite for the enhanced electrochemical sensitivity towards urea oxidation. J. Alloys Compd..

[29] Ashwini R., Punith Kumar M.K., Rekha M.Y., Santosh M.S., Srivastava C. (2022). High entropy alloy nanoparticle–graphene (HEA:G) composite for non-enzymatic glucose oxidation: Optimization for enhanced catalytic performance. Carbon Trends.

[30] Zhao D., Yu G., Tian K., Xu C. (2016). A highly sensitive and stable electrochemical sensor for simultaneous detection towards ascorbic acid, dopamine, and uric acid based on the hierarchical nanoporou sPtTi alloy. Biosens. Bioelectron..

[31] Jin L., Zhang Z., Zhuang Z., Meng Z., Li C., Shen Y. (2016). PdPt bimetallic alloy nanowires-based electrochemical sensor for sensitive detection of ascorbic acid. RSC Adv..

[32] Shashanka R., Chaira D., Swamy B.E.K. (2015). Electrochemical investigation of duplex stainless steel at carbon paste electrode and its application to the detection of dopamine, ascorbic and uric acid. Int. J. Sci. Eng. Res..

[33] Shashanka R., Chaira D., Swamy B.E.K. (2016). Fabrication of yttria dispersed duplex stainless steel electrode to determine dopamine, ascorbic and uric acid electrochemically by using cyclic voltammetry. Int. J. Sci. Eng. Res..

[34] Shashanka R., Chaira D. (2015). Optimization of milling parameters for the synthesis of nano-structured duplex and ferritic stainless steel powders by high energy planetary milling. Powder Technol..

[35] Nayak A.K., Shashanka R., Chaira D. (2016). Effect of Nanosize Yittria and Tungsten Addition to Duplex Stainless Steel During High Energy Planetary Milling. IOP Conf. Ser. Mater. Sci. Eng..

[36] Shashanka R. (2017). Synthesis of nano-structured stainless steel powder by mechanical alloying-an overview. Int. J. Sci. Eng. Res..

[37] Shashanka R. (2019). Non-lubricated dry sliding wear behavior of spark plasma sintered nano-structured stainless steel. J. Mater. Environ. Sci..

[38] Ashoka N.B., Swamy B.E.K., Jayadevappa H. (2017). Nanorod TiO_2_ sensor for dopamine: A voltammetric study. New J. Chem..

[39] Rajendrachari S., Kudur Jayaprakash G., Pandith A., Karaoglanli A.C., Uzun O. (2022). Electrocatalytic Investigation by Improving the Charge Kinetics between Carbon Electrodes and Dopamine Using Bio-Synthesized CuO Nanoparticles. Catalysts.

[40] Charithra M.M., Manjunatha J.G., Prinith N.S., Pushpanjali P.A., Girish T., Hareesha N. (2022). Electroanalytical Determination of Tinidazole by using Surface Modified Carbon Nano Composite based Sensor. Mater. Res. Innov..

[41] Charithra M.M., Manjunatha J.G. (2019). Poly (L-Proline) modified carbon paste electrode as the voltammetric sensor for the detection of Estriol and its simultaneous determination with Folic and Ascorbic acid. Mater. Sci. Energy Technol..

[42] Hareesha N., Manjunatha J.G., Raril C., Tigari G. (2019). Sensitive and Selective Electrochemical Resolution of Tyrosine with Ascorbic Acid through the Development of Electropolymerized Alizarin Sodium Sulfonate Modified Carbon Nanotube Paste Electrodes. ChemistrySelect.

[43] Prinith N.S., Manjunatha J.G., Raril C. (2019). Electrocatalytic Analysis of Dopamine, Uric Acid and Ascorbic Acid at Poly(Adenine) Modified Carbon Nanotube Paste Electrode: A Cyclic Voltammetric Study. Anal. Bioanal. Electrochem..

[44] Raril C., Manjunatha J.G., Ravishankar D.K., Fattepur S., Siddaraju G., Nanjundaswamy L. (2020). Validated Electrochemical Method for Simultaneous Resolution of Tyrosine, Uric Acid, and Ascorbic Acid at Polymer Modified Nano-Composite Paste Electrode. Surf. Eng. Appl. Electrochem..

